# Mutational analysis of a phenazine biosynthetic gene cluster in *Streptomyces anulatus* 9663

**DOI:** 10.3762/bjoc.8.57

**Published:** 2012-04-04

**Authors:** Orwah Saleh, Katrin Flinspach, Lucia Westrich, Andreas Kulik, Bertolt Gust, Hans-Peter Fiedler, Lutz Heide

**Affiliations:** 1 Pharmaceutical Institute, University of Tübingen, Auf der Morgenstelle 8, 72076 Tübingen, Germany; 2 Institute of Microbiology and Infection Medicine, University of Tübingen, Auf der Morgenstelle 28, 72076 Tübingen, Germany

**Keywords:** phenazine, gene cluster, gene inactivation

## Abstract

The biosynthetic gene cluster for endophenazines, i.e., prenylated phenazines from *Streptomyces anulatus* 9663, was heterologously expressed in several engineered host strains derived from *Streptomyces coelicolor* M145. The highest production levels were obtained in strain M512. Mutations in the *rpoB* and *rpsL* genes of the host, which result in increased production of other secondary metabolites, had no beneficial effect on the production of phenazines. The heterologous expression strains produced, besides the known phenazine compounds, a new prenylated phenazine, termed endophenazine E. The structure of endophenazine E was determined by high-resolution mass spectrometry and by one- and two-dimensional NMR spectroscopy. It represented a conjugate of endophenazine A (9-dimethylallylphenazine-1-carboxylic acid) and L-glutamine (L-Gln), with the carboxyl group of endophenazine A forming an amide bond to the α-amino group of L-Gln. Gene inactivation experiments in the gene cluster proved that *ppzM* codes for a phenazine *N*-methyltransferase. The gene *ppzV* apparently represents a new type of TetR-family regulator, specifically controlling the prenylation in endophenazine biosynthesis. The gene *ppzY* codes for a LysR-type regulator and most likely controls the biosynthesis of the phenazine core. A further putative transcriptional regulator is located in the vicinity of the cluster, but was found not to be required for phenazine or endophenazine formation. This is the first investigation of the regulatory genes of phenazine biosynthesis in *Streptomyces*.

## Introduction

Phenazine natural products have important biological activities comprising antibacterial, antifungal, antitumor, antimalarial, antioxidant and antiparasitic activities, and as inhibitors of angiotensin converting enzyme (ACE) or testosterone-5-α-reductase [1]. The synthetic phenazine clofazimine has been approved for human therapy in the treatment of leprosy. Some of the other naturally occurring phenazines are bacterial virulence factors [1]. Natural phenazines are secondary metabolites, produced mainly by different species of the proteobacterium *Pseudomonas* and of the actinobacterium *Streptomyces*. While *Pseudomonas* strains produce phenazine derivatives with relatively simple structures, more complex phenazines are produced by *Streptomyces* strains [1]. The biosynthesis of phenazine-1-carboxylic acid (PCA) and its derivatives has been studied extensively in *Pseudomonas* [2–5]. The biosynthesis of PCA requires a set of seven genes named *phzABCDEFG* [3,6]. PhzC codes for DAHP (3-deoxy-D-arabinoheptulosonate-7-phosphate) synthase, the first enzyme of the shikimate pathway, and ensures the flow of primary metabolites towards chorismic acid. Chorismic acid is the branch point at which the biosynthesis of PCA, catalyzed by the enzymes PhzABDEFG, branches off from the shikimate pathway. These seven core phenazine biosynthesis genes could be identified in nearly all investigated bacterial strains that produce phenazine compounds [3,6]. Other genes have been shown to play a role in the regulation of phenazine biosynthesis. In *P. fluorescens*, the transcriptional regulation involves the quorum sensing proteins PhzR/PhzI, the positive two-component regulator system GacS/GacA, and the negative two-component regulator system RpeA/RpeB [7–8]. Additional regulatory genes have been identified in *P. chlororaphis*, including the transcriptional regulator gene *pip* and post-transcriptional regulators encoded by *rsmA* and *rsmZ* [7,9–10]. Although many different phenazines are produced by *Streptomyces* strains, only two gene clusters have been identified in *Streptomyces* so far, i.e., the phenazine biosynthetic gene clusters from *S. anulatus* [11] and from *S. cinnamonensis* [12–13]. In *Streptomyces*, it is as yet completely unknown which genes are involved in the regulation of the biosynthesis of phenazine natural products.

**Figure 1:**
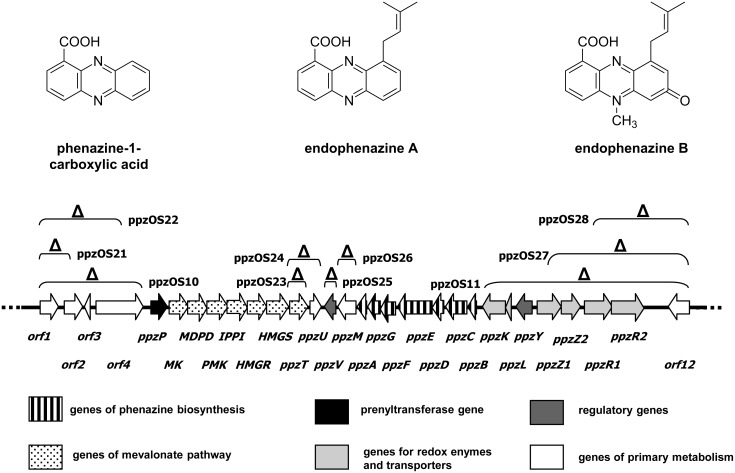
The endophenazine biosynthetic gene cluster from *Streptomyces anulatus* 9663 and the structures of phenazine-1-carboxylic acid and endophenazines A and B. The depicted sequence corresponds to the insert of cosmid ppzOS04. The gene deletions carried out in this study and the names of the resulting constructs are indicated.

In a previous study, we described the biosynthetic gene cluster for prenylated phenazines from *Streptomyces anulatus* (Figure 1) [11]. This cluster contained the seven core phenazine biosynthesis genes, the mevalonate pathway genes and a prenyltransferase gene, and further genes with unknown functions. Heterologous expression of this cluster, contained in cosmid ppzOS04, in *Streptomyces coelicolor* M512 yielded similar phenazine compounds as formed by the wild-type producer strain, with PCA and endophenazine A as the dominant compounds, and endophenazine B as a minor product (Figure 1) [11].

In the present study, we carried out inactivation experiments of genes on cosmid ppzOS04, followed by heterologous expression of the modified clusters and chemical analysis of secondary metabolite formation. This allowed us to investigate the function of individual genes of this cluster for the biosynthetic pathway and for its regulation. The genes inactivated in this study are summarized in Table 1, and a complete list of the genes contained in the insert of cosmid ppzOS04 is given in Table S1 of Supporting Information File 1.

**Table 1:** Genes investigated in this study.

gene	aa	proposed function	orthologue identified by BLAST search	identity/ similarity %	acc. number

*orf1*	333	serine protease	putative serine protease, *Streptomyces roseosporus* NRRL 15998	93/98	ZP_04708306
*orf2*	342	aspartate-semialdehyde dehydrogenase	ASD2*, Streptomyces griseus* subsp. griseus NBRC 13350	97/99	YP_001826399
*orf3*	115	putative transcriptional modulator	*Streptomyces roseosporus* NRRL 11379	71/87	ZP_04708308
*orf4*	870	aminopeptidase N	*Streptomyces griseus* subsp. griseus NBRC 13350	98/99	YP_001826397
(prenyltransferase gene *ppzP* and six genes of the mevalonate pathway)					
*ppzT*	327	putative acetoacetyl-CoA synthase	*Streptomyces* sp. KO-3988	79/87	BAD86806
*ppzU*	221	flavodoxin	flavoprotein WrbA, *Streptomyces violaceusniger* Tü 4113	63/79	YP_004814680
*ppzV*	206	putative TetR-family regulator	EpzV, *Streptomyces cinnamonensis*	64/76	ADQ43382
*ppzM*	340	*N*-Methyltransferase	EpzM, *Streptomyces cinnamonensis*	77/86	ADQ43384
(genes *ppzBCDEFGA* of phenazine-1-carboxylic acid biosynthesis)					
*ppzK*	419	FAD-dependent oxidoreductase	FAD-dependent pyridine nucleotide-disulfide oxidoreductase	55/66	YP_295688
*ppzL*	107	ferredoxin	ferredoxin, *Rhodopseudomonas palustris* HaA2	55/68	YP_487220
*ppzY*	290	transcriptional regulator	transcriptional regulator, *Streptomyces lividans* TK24	61/74	ZP_06530228
*ppzZ1*	430	cytochrome d ubiquinol oxidase, subunit I	cytochrome d ubiquinol oxidase, subunit I, *Stackebrandtia nassauensis* DSM 44728	54/66	YP_003509915
*ppzZ2*	344	cytochrome d ubiquinol oxidase, subunit II	cytochrome d ubiquinol oxidase, subunit II, *Stackebrandtia nassauensis* DSM 44728	48/60	YP_003509914
*ppzR1*	563	ABC transporter	cysteine ABC transporter permease, *Thermobispora bispora* DSM 43833	55/68	YP_003653341
*ppzR2*	585	ABC transporter	cysteine ABC transporter permease, *Streptosporangium roseum* DSM 43021	52/65	YP_003338959
*orf12*	384	allantoicase	putative allantoicase, *Streptomyces griseus* subsp. griseus NBRC 13350	98/99	YP_001826394

## Results and Discussion

### Production of prenylated phenazines by cultivation of the heterologous producer strain in 24 square deep-well plates

One important aspect of the current study was the investigation of the influence of putative regulatory genes on the production of endophenazines. Therefore, it was important to assess quantitative differences in production reliably. We decided to use cultivation in 24 square deep-well plates (EnzyScreen BV, The Netherlands). Previous studies have shown that this greatly reduces the variability of secondary metabolite production in comparison to cultivation in Erlenmeyer flasks [14]. In order to obtain a uniform inoculum, precultures were harvested at a defined growth stage, i.e., before reaching the stationary phase. The mycelia were finely dispersed by brief treatment with a Potter homogenizer, frozen in the presence of peptone and stored at −70 °C. Aliquots of this inoculum were used to inoculate individual wells of the deep-well plates, with each well containing 3 mL medium. In initial experiments, the medium was supplemented with 0.6% (w/v) of the siloxylated ethylene oxide/propylene oxide copolymer Q2-5247 (Dow Corning, USA), which acts as an oxygen carrier and has been shown to increase the production of certain antibiotics [14].

Of each mutant obtained in this study, usually three independent clones were isolated, and secondary metabolite production was determined in three parallel cultivations for each clone. The variability of production between different clones, and between parallel cultivations of the same clone, was relatively low (average standard deviation of 19.2%).

### Expression of the endophenazine gene cluster in different host strains

**Figure 2:**
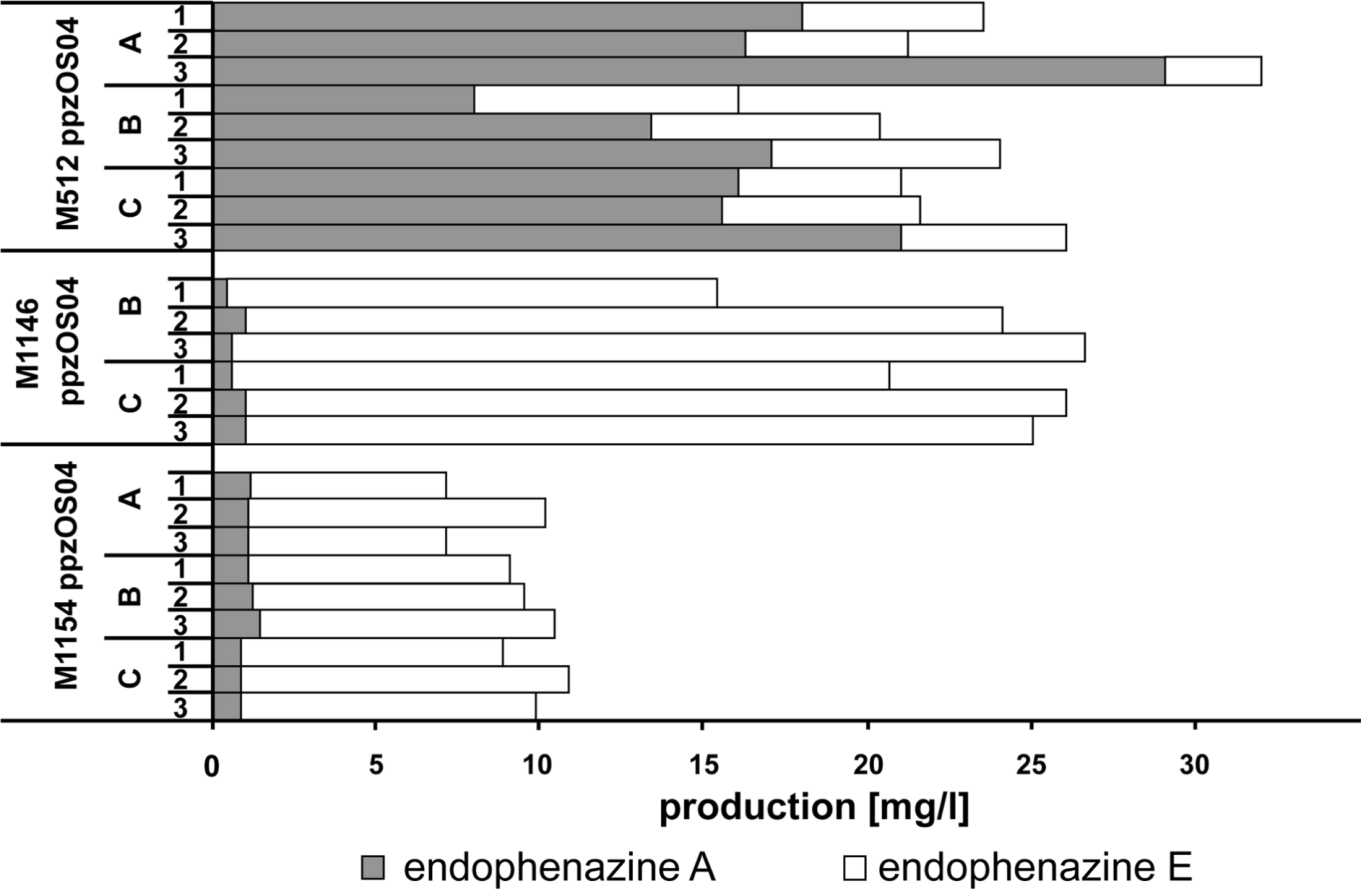
Production of prenylated phenazines after heterologous expression of the endophenazine gene cluster in different expression hosts. From each expression host, two to three independent clones were obtained (A–C), and production was determined in three parallel cultivations of each clone (1–3). Cultivation was carried out in 24 square deep-well plates. In the experiments depicted here, the culture medium was supplemented with 0.6% of the oxygen carrier Q2-5247.

Previous heterologous expression experiments of the endophenazine gene cluster have been carried out using *Streptomyces coelicolor* M512 as a host strain [11]. Recently, new heterologous expression strains were generated from *S. coelicolor* M145, the parental strain of M512. These new strains include M1146, in which the entire biosynthetic gene clusters of actinorhodin, undecylprodigiosine and calcium-dependent antibiotic, as well as the so-called *cpk* cluster, have been deleted, and which also lacks plasmids SCP1 and SCP2. Furthermore, strain M1154 was generated from strain M1146 by introducing mutations into the genes *rpoB* and *rpsL*, which has been shown to result in an increased production of certain antibiotics [15]. We have now introduced cosmid ppzOS04, which contains the entire gene cluster of the endophenazines [11], into these two strains. However, as depicted in Figure 2, the highest production was achieved in strain M512. Therefore, the *rpoB* and *rpsL* mutations, and the deletion of the other biosynthetic gene cluster, have no beneficial effect on the production of phenazines, and all further experiments in this study were carried out by using M512 as the heterologous expression strain.

### Identification of a new phenazine natural product

**Figure 3:**
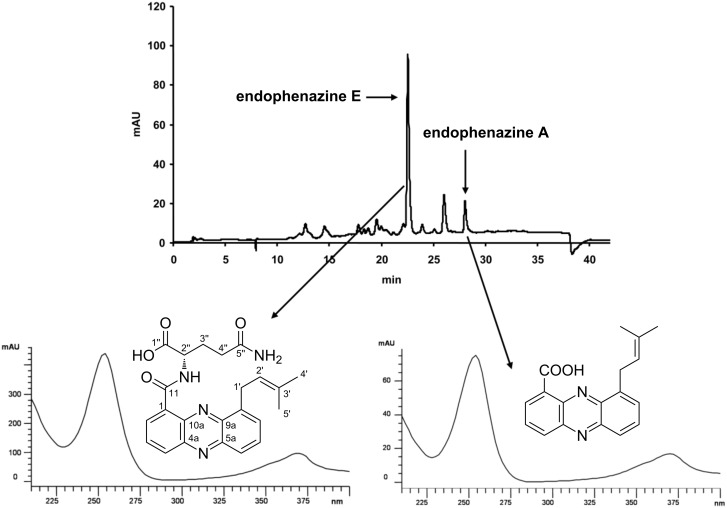
HPLC analysis of mycelia of the heterologous expression strain *S. coelicolor* M512(ppzOS04) after five days of cultivation. The oxygen carrier Q2-5247 was not included in the culture medium in this experiment. Detection wavelength: 365 nm. The lower panels show the UV spectra of endophenazine A and endophenazine E.

As shown in Figure 2 and Figure 3, heterologous expression of cosmid ppzOS04 did not only result in the formation of endophenazine A, but also of another compound with the typical absorption spectrum of phenazines. This compound was termed endophenazine E. In M1146 and M1154, endophenazine E was the dominant product in all investigated samples. In M512, endophenazine E was a minor compound after five days of cultivation in the presence of the oxygen carrier Q2-5247 (Figure 2). In the absence of Q2-5247, endophenazine E was a minor compound after three days of cultivation, but became the dominant compound after five days. The time course of the formation of endophenazine A and E during seven days of cultivation is depicted in Figure S1 (Supporting Information File 1).

Endophenazine E showed a molecular ion at *m*/*z* 421 ([M + H]^+^). Positive-ion-mode high-resolution mass spectrometry showed an exact mass of 421.186790 Dalton, indicating a molecular formula of C_23_H_24_N_4_O_4_ (calculated mass 420.1870317 Dalton, Δ = 0.57 ppm), different from any phenazine derivative described previously.

To identify the structure of the new product, the heterologous expression strain *S. coelicolor* M512(ppzOS04) carrying the phenazine biosynthetic gene cluster was cultivated in a 10 L fermenter. Endophenazine E was purified from the mycelia by chromatography on Sephadex LH-20 and by preparative reversed-phase HPLC. Sixty milligrams of a yellow solid compound was obtained and 7 mg were investigated by unidimensional (^1^H and ^13^C) and multidimensional (COSY, HSQC and HMBC) NMR spectroscopy, in comparison to PCA. This showed signals for a phenazine core and for a prenyl group, very similar to those shown by endophenazine A. The additional signals showed that the carboxyl group of endophenazine A was attached to the α-amino group of the amino acid glutamine. The ^1^H and ^13^C NMR data of the compound are summarized in Table 2, and the ^1^H-^1^H COSY, HSQC and HMBC correlations are depicted in Figure S2 (Supporting Information File 1).

**Table 2:** Full ^1^H and ^13^C NMR spectroscopic data of endophenazine E. Chemical shifts are expressed in δ values with the solvent as the internal standard.

position	^13^C NMR data (100.6 MHz, MeOD) δ_C_ [ppm]	^1^H NMR data (400 MHz, MeOD) δ_H_ [ppm]	^1^H NMR data (400 MHz, *d*_6_-DMSO) δ_H_ [ppm]

1	130.0	—	—
2	136.3	8.83, 1H, dd, *J* = 7.2, 1.4 Hz	8.80, 1H, dd, *J* = 7.2, 1.5 Hz
3	131.1	8.01, 1H, dd, *J* = 8.7, 7.2 Hz	8.10, 1H, dd, *J* = 8.7, 7.2 Hz
4	135.0	8.38, 1H, dd, *J* = 8.7, 1.4 Hz	8.46, 1H, dd, *J* = 8.7, 1.5 Hz
4a	144.0	—	—
5a	144.6	—	—
6	128.3	8.06, 1H, bd, *J* = 8.7 Hz	8.15, 1H, dd, *J* = 8.1, 0.7 Hz
7	132.9	7.87, 1H, dd, *J* = 8.7, 6.8 Hz	7.95, 1H, dd, *J* = 8.7, 6.7 Hz
8	131.3	7.75, 1H, dd, *J* = 6.8, 1.0 Hz	7.80, 1H, dd, *J* = 6.7, 0.7 Hz
9	141.7	—	—
9a	141.9	—	—
10a	140.9	—	—
11	166.8	—	—
1′	30.8	4.17, 2H, d, *J* = 7.3 Hz	4.13, 2H, dd, *J* = 7.2, 7.2 Hz
2′	122.4	5.58, 1H, dddd, *J* = 7.3, 7.3, 1.0, 1.0 Hz	5.57, 1H, dddd, *J* = 7.2, 7.2, 1.2, 1.2 Hz
3′	135.7	—	—
4′	26.0	1.82, 3H, bs	1.73, 3H, bs
5′	18.1	1.80, 3H, bs	1.73, 3H, bs
1″	174.8	—	—
2″	54.0	4.96, 1H, dd, *J* = 9.1, 4.1 Hz	4.74, ddd, 1H, *J* = 8.7, 8.7, 3.9 Hz
2″-NH			10.9, d, *J* = 8.7 Hz
3″	29.8	2.18–2.30, 1H_a_, m 2.16–2.36, 1H_b_, m	1.93–2.09, 1H_a_, m 2.22–2.38, 1H_b_, m
4″	33.2	2.16–2.36, 2H, m	2.22–2.38, 2H, m
5″	177.4	—	—
5″-NH_2_		—	6.73, 1H_a_, bs 7.27, 1H_b_, sb

The configuration at the α-carbon of the amino acid was determined as L-Gln by enantioselective HPLC analysis [16] (see Experimental section). The specific rotation was determined as 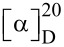 = +16.8 (*c* = 0.33, MeOH).

Endophenazine E is a new natural product. The conjugation of a phenazine to *N-*acetylcysteine has been described previously [17]. In that case, conjugation occurred through the thiol group of cysteine and led to the loss of the antibacterial activity of the phenazine. A similar *N-*acetylcysteine adduct has been described for a polyketide antibiotic, also leading to a loss of biological activity; therefore, the conjugation has been suggested as representing a detoxification mechanism [18].

The extent of the conversion of endophenazine A to endophenazine E in cultures of *S. coelicolor* M512(ppzOS04) depended on the cultivation conditions. Only small amounts of endophenazine E are formed by cultivation in Erlenmeyer flasks. Upon cultivation in 24 square deep-well plates, endophenazine E is still a minor compound if the oxygen supply is improved by the inclusion of the oxygen carrier Q2-5247. If Q2-5247 is omitted from the medium, however, endophenazine E becomes the dominant product once the culture has reached the stationary growth phase. Q2-5247 did not affect the total amount of prenylated phenazines formed, and we omitted it from the culture medium in all subsequent experiments.

The conversion of endophenazine A to endophenazine E was almost complete in strains M1146 and M1154, in contrast to strain M512 (Figure 2). In the former two strains, the entire biosynthetic gene clusters of actinorhodin, undecylprodigiosine and calcium-dependent antibiotic as well as the so-called *cpk* cluster have been deleted. However, it is unknown how these deletions are connected to the conversion of endophenazine A to endophenazine E.

### Determination of the borders of the endophenazine cluster

The left side of the endophenazine gene cluster depicted in Figure 1 contains the phenazine prenyltransferase gene *ppzP* and, downstream thereof, the genes of the mevalonate pathway for supply of the isoprenoid precursor dimethylallyl diphosphate (DMAPP). Upstream of *ppzP*, four genes, *orf1-orf4,* could be identified. Database comparisons by using BLAST and Pfam searches gave no obvious clues as to whether or not they are involved in the biosynthesis of secondary metabolites. The gene *orf1* (1002 bp) showed similarities to serine proteases, and *orf2* (1029 bp) to aspartate-semialdehyde dehydrogenases. The gene *orf3* (348 bp) showed homology to transcriptional modulators of the PemK-like protein family [19]; PemK binds to the promoter region of the Pem operon in *E. coli.* Finally, *orf4* (2613 bp) showed homology to aminopeptidases.

λ-RED recombination was used to delete the entire coding sequence of *orf1* and the first 437 nucleotides of *orf2* from the insert of cosmid ppzOS04 (Figure 1). After recombination of the pIJ773 cassette harbouring an apramycin resistance gene, the disruption cassette was excised by FLP recombinase. The correct sequence of the resulting cosmid ppzOS21 was confirmed by restriction analysis and PCR. Cosmid ppzOS21 was introduced into *S. coelicolor* M512 by triparental mating, and stably integrated into the *attB* site of the genome. Three independent integration mutants were obtained, and their secondary metabolite production was investigated by HPLC in comparison to a strain harbouring the unmodified cosmid ppzOS04 (Table 3). Both strains produced similar amounts of prenylated phenazines (162 or 201 µmol/L, respectively), besides smaller amounts of nonprenylated phenazines. This proved that *orf1* and *orf2* are not required for the formation of endophenazines.

**Table 3:** Production of nonprenylated and of prenylated phenazines in the heterologous expression strain *Streptomyces coelicolor* M512, carrying either the complete endophenazine gene cluster (ppzOS04) or clusters in which individual genes had been deleted. Data represent mean values ±SD.

integrated construct	description	nonprenylated phenazines [µmol/L] (phenazine-1-carboxylic acid and phenazine-1-carboxylic acid methyl ester)	prenylated phenazines [µmol/L] (endophenazine A, B and E)

ppzOS04	complete cluster	17.8 ± 4.0	200.8 ± 42.7
ppzOS21	Δ(*orf1* − *orf2*)	6.7 ± 2.4	161.5 ± 35.9
ppzOS10	Δ(*orf1* − *orf4*)	54.9 ± 4.2	23.3 ± 1.6
ppzOS22	Δ(*orf1* − middle of *orf4*)	12.4 ± 1.9	112.8 ± 20.4
ppzOS23	Δ*ppzT*	7.3 ± 1.6	103.9 ± 16.8
ppzOS24	Δ(*ppzT* + *ppzU*)	6.1 ± 1.7	2.4 ± 0.9
ppzOS25	Δ*ppzV*	58.3 ± 21.0	<0.1
ppzOS26	Δ*ppzM*	4.5 ± 0.5	165.5 ± 60
ppzOS11	Δ(*ppzK* − *orf12*)	1.1 ± 0.3	0.4 ± 0.2
ppzOS27	Δ(*ppzZ1* − *orf12*)	17.5 ± 3.5	3.1 ± 1.07
ppzOS28	Δ(*ppzR1* − *orf12*)	14.8 ± 5.2	31.4 ± 5.1

However, when we deleted a DNA fragment comprising all four genes from *orf1* to *orf4* by the same procedure, the resulting strain, i.e., *S. coelicolor* M512(ppzOS10) showed a strongly reduced formation of prenylated phenazines (23 µmol/L). At the same time, the production of nonprenylated phenazines was increased, indicating that the mutation affected the formation or attachment of the prenyl moiety of the endophenazines. Two alternative hypotheses may explain this observation: The regulatory gene *orf3* may be involved in the regulation of the prenylation; or the deletion of the entire *orf4* sequence may have affected the promoter of the prenyltransferase gene *ppzP*, which is situated downstream of *orf4*. In order to distinguish between these two possibilities, an additional λ-RED-mediated gene inactivation was carried out (Figure 1). The genes *orf1*, *orf2, orf3* and the first 1092 bp of *orf4* were deleted, resulting in cosmid ppzOS22. In this construct, the regulator *orf3* is deleted, but the promoter region of *ppzP* is expected to be intact, even if it would extend into the coding sequence of *orf4*. Integration of this cosmid into the heterologous expression host resulted in a strain, which again produced endophenazines (113 µmol/L) (Table 3). These results show that *orf3* and *orf4* are not essential for the production of prenylated phenazines. The strong reduction of prenylated phenazine production in mutant *S. coelicolor*(ppzOS10) is most likely due to the absence of *ppzP* promoter sequences located within the coding sequence of the gene *orf4*. In a previous study, we have already shown that deletion of the prenyltransferase gene *ppzP* results in a complete abolishment of the production of prenylated phenazines [11]. Therefore, *ppzP* (including its promoter region) may represent the left border of this cluster.

The right side of the endophenazine gene cluster depicted in Figure 1 contains the operon of phenazine biosynthesis genes, *ppzBCDEFGA*, oriented in the opposite direction to the mevalonate biosynthesis genes. Upstream of *ppzB*, seven genes are found (*ppzK* to *ppzR2*), the function of which is unclear. Orthologues for these seven genes, arranged in exactly the same order and orientation, were also identified next to the endophenazine gene cluster of *S. cinnamonensis* DSM 1042 [12].

The gene *ppzK* shows similarities to FAD-dependent oxidoreductases, and *ppzL* to ferredoxin. The gene *ppzY* is similar to the transcriptional regulator SCO3435 of *Streptomyces coelicolor* A3(2). The genes *ppzZ1* and *ppzZ2* have similarities to the two subunits of cytochrome d ubiquinol oxidase, and *ppzR1* and *ppzR2* to ABC transporters. The next gene, *orf12*, shows very high similarities to the primary metabolic enzyme allantoicase (allantoate amidohydrolase, EC 3.5.3.4), an enzyme of purine catabolism. It is separated from *ppzR2* by a gap of 1.3 kb and oriented in the opposite direction.

In order to determine the border of the gene cluster, we carried out three parallel inactivation experiments, in which either all genes from *ppzK* to *orf12*, or the genes from *ppzZ1* to *orf12*, or only the genes from *ppzR1* to *orf12* were deleted from cosmid ppzOS04, by using λ-RED recombination and the same procedure as described above. Analysis of the secondary metabolite production in the Δ*ppzR1-orf12* mutant showed that the formation of prenylated phenazines was reduced to 31 µmol/L, i.e., to approximately 17% of the amount formed in the strain with the intact cosmid; production of nonprenylated phenazines was similar in both strains. The most likely explanation of this result is that the ABC transporters encoded by *ppzR1* and *ppzR2* are involved in the export of endophenazines, and therefore their inactivation reduces but does not completely prevent the production of these compounds. In the Δ*ppzZ1-orf12* mutant, the production of prenylated phenazines was reduced even further (to 3.1 µmol/L), while the production of nonprenylated phenazines was similar to that of the strain with the intact cluster (17.5 µmol/L). This suggests that *ppzZ1* and *ppzZ2*, encoding proteins similar to the two subunits of prokaryotic cytochrome d ubiquinol oxidase, play a role in the formation of prenylated phenazines. Cytochrome d ubiquinol oxidase reduces ubiquinone to ubiquinol, a reaction similar to the reduction of PCA to 5,10-dihydro-PCA (Figure S3, Supporting Information File 1). The compound 5,10-dihydro-PCA, but not PCA, is the substrate for prenylation by the prenyltransferase PpzP [11]. This may explain why the deletion of *ppzZ1*-*ppzZ2* resulted in a reduced formation of prenylated phenazines.

In the Δ*ppzK-orf12* mutant, finally, the production of both prenylated and nonprenylated phenazines was nearly abolished. A possible explanation is that the putative regulatory gene *ppzY* plays a role in the regulation of phenazine biosynthesis. PpzY is similar to transcriptional regulators of the LysR family, the most abundant type of transcriptional regulators in the prokaryotic kingdom [20]. The LysR-like protein PqsR from *Pseudomonas* sp. M18 is involved in the regulation of phenazine biosynthesis. Inactivation of *pqsR* resulted in almost complete abolishment of the transcription of the phenazine biosynthesis genes [21]. It may therefore be speculated that *ppzY* codes for a positive regulator of PCA biosynthesis.

Based on these results, the large intergenic region between the ABC transporter gene *ppzR2* and the primary metabolic gene *orf12* (allantoicase) is likely to represent the right border of the endophenazine cluster.

### Functional investigation of the four genes *ppzTUVM*, situated in the center of the endophenazine cluster

**Figure 4:**
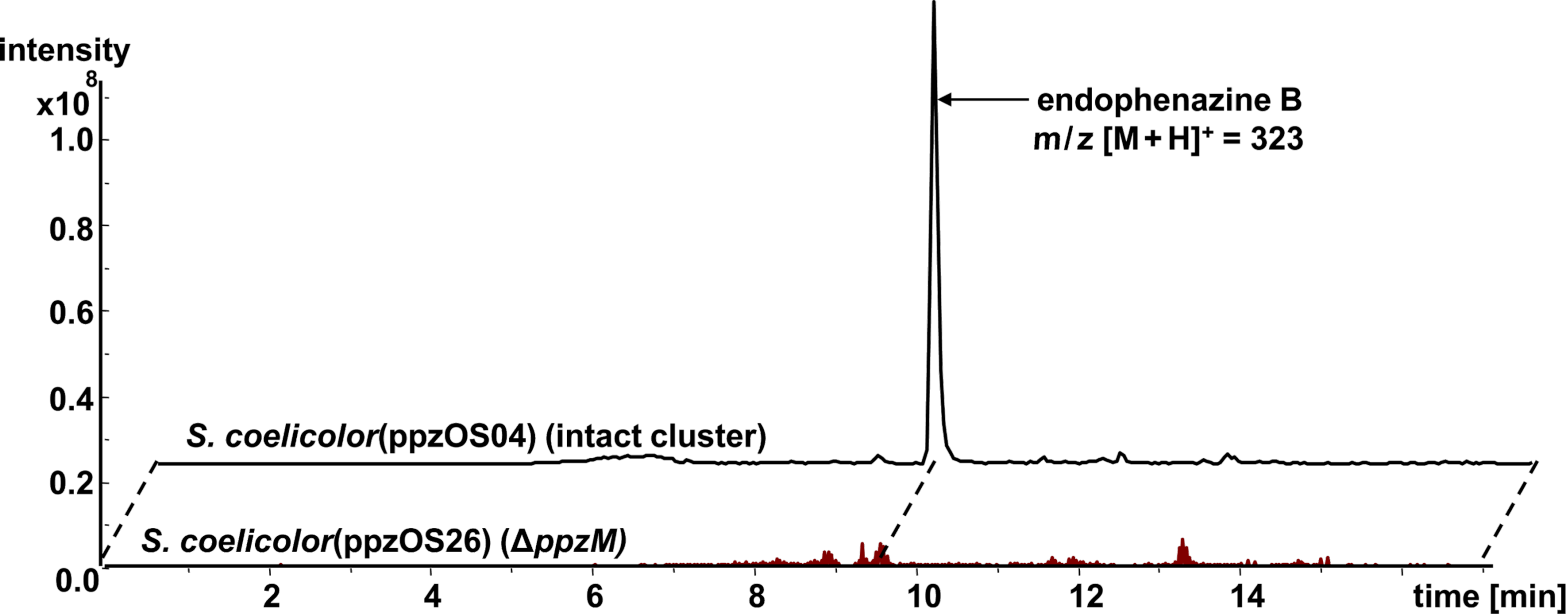
Extracted ion chromatograms for the mass of endophenazine B (*m*/*z* [M + H]^+^ = 323) in *S. coelicolor* M512(ppzOS04), and the mutant *S. coelicolor* M512(ppzOS26). The deletion of the gene *ppzM* reveals the abolishment of the production of endophenazine B.

In between the mevalonate pathway genes and the dihydro-PCA biosynthesis genes, four further genes are situated, i.e., *ppzTUVM* (Figure 1). The gene *ppzM* (1023 bp) shows similarities to PhzM from *Pseudomonas aeruginosa* PAO1, a putative phenazine-specific methyltransferase that catalyzes the *N*-methylation reaction during the biosynthesis of pyocyanine in *Pseudomonas*. Also, *Streptomyces anulatus* produces an *N*-methylated phenazine, i.e., endophenazine B [22]. The heterologous expression strain *S. coelicolor* M512 containing the endophenazine cluster from *S. anulatus* produces very low amounts of endophenazine B, such that detection requires LC-MS analysis (Figure 4). We deleted *ppzM* using λ-RED recombination and the resulting construct, ppzOS26, was introduced into *S. coelicolor* M512. HPLC-UV analysis of cultures of the resulting strain showed a similar production of prenylated and nonprenylated phenazines to that observed in the strain with the intact cluster (Table 3). HPLC-ESI-MS analysis, however, revealed that the production of endophenazine B had been abolished by the *ppzM* deletion (Figure 4 and Figure S4, Supporting Information File 1). The gene *ppzM* is thereby the first phenazine *N*-methyltransferase gene identified in *Streptomyces*.

We expressed the protein PpzM in *E. coli* and purified it using Ni^2+^ affinity chromatography. However, incubation of PpzM with *S*-adenosylmethionine and either PCA or dihydro-PCA did not result in the formation of any methylated derivatives. It has been reported that, likewise, PhzM from *P. aeroginosa* was not active when incubated with PCA and SAM alone; methylating activity was only detected when the hydroxylase PhzS and its cofactor NADH were also included in the incubation [23–24]. The endophenazine biosynthetic gene cluster from *S. anulatus* does not contain an orthologue of PhzS*.* Possibly, PpzM requires association with another protein for its activity, but it cannot be decided at present which protein this may be.

The function of *ppzT* (984 bp) was unknown when we first published the sequence of the endophenazine gene cluster [11]. The predicted protein PpzT showed similarities to putative 3-oxoacyl-[acyl-carrier-protein] synthases. Recently, Okamura et al. [25] proved that *nphT7*, a gene that is highly similar to *ppzT*, is responsible for catalysing the first step of the mevalonate pathway in naphterpin biosynthesis, i.e., the biosynthesis of acetoacetyl-CoA from malonyl-CoA and acetyl-CoA. In many other organisms, acetoacetyl-CoA for mevalonate biosynthesis is produced by a different reaction, i.e., by acetyl-CoA acetyltransferase (= acetoacetyl-CoA thiolase). We deleted *ppzT* from cosmid ppzOS04 and heterologously expressed the resulting cosmid ppzOS23. Analysis of the obtained strain showed that the formation of prenylated phenazines was reduced by approximately 50% in comparison to the strain containing the intact cluster. Since the heterologous host strain *S. coelicolor* M512 contains several putative acetyl-CoA acetyltransferase genes, which may generate acetoacetyl-CoA, a reduction, but not an abolishment, of the formation of prenylated phenazines was indeed the expected result after inactivation of *ppzT*. It should also be noted that *Streptomycetes* synthesize DMAPP for primary metabolism via the nonmevalonate pathway (MEP pathway), enabling a further route to prenylated phenazines in the Δ*ppzT* mutant [26].

The gene *ppzU* (348 bp) shows high similarity to flavodoxin from *Streptomyces roseosporus* NRRL 15998 and to the closely related flavoprotein WrbA from *Streptomyces flavogriseus* ATCC 33331. Flavodoxins are mobile electron carriers containing a flavin mononucleotide as the prosthetic group and mediating redox processes among a promiscuous set of donors and acceptors. For unknown reasons, the individual deletion of *ppzU* from cosmid ppzOS04 was unsuccessful. However, we did succeed in the deletion of the DNA fragment comprising both *ppzT* and *ppzU,* resulting in cosmid ppzOS24. Strains expressing this cosmid showed an almost complete abolishment of the production of prenylated phenazines, while the production of nonprenylated phenazine derivatives was not different from the Δ*ppzT* single mutant (Table 3). This suggests that *ppzU* plays a role for the prenylation in endophenazine biosynthesis. For instance, it could act as an electron carrier for the above-mentioned heterodimeric redox enzyme PpzZ1/PpzZ2, which may catalyze the reduction of PCA to 5,10-dihydro-PCA and thereby generate the substrate of the prenyltransferase PpzP. In *E. coli*, WrbA has been suggested to carry out a regulatory function in addition to its biochemical function [27], but this hypothesis is controversial [28].

The gene *ppzV* (621 bp) shows similarities in BLAST searches to *ovmZ*, a gene with unknown function in the biosynthetic gene cluster of oviedomycin from *Streptomyces antibioticus* [29]. Orthologs of *ovmZ* are found in the gene clusters of several prenylated secondary metabolites, e.g., *fnq22* in the furanonaphthoquinone/phenazine biosynthesis gene cluster from *Streptomyces cinnamonensis* [13], *napU1* in the napyradiomycin biosynthesis gene cluster from *S. aculeolatus* [30]*,* and *fur18* in the biosynthetic gene cluster of furaquinocin A from *Streptomyces* sp. KO-3988 [31]. A similar gene, *aur1O*, is found in the biosynthetic gene cluster of the polyketide antibiotic auricin from *S. aureofaciens* [32]. The function of these proteins is unknown. Pfam searches do not show any match to known protein domains. Therefore, the possible function of *ppzV* was obscure. We deleted *ppzV* using λ-RED recombination and the resulting construct ppzOS25 was introduced into *S. coelicolor* M512. Unexpectedly, the resulting strains showed a nearly complete abolishment of the formation of prenylated phenazines, while the production of nonprenylated phenazines increased (Table 3). This suggests that *ppzV* inactivation had drastically and specifically affected the prenylation step in endophenazine biosynthesis. This prompted us to carry out further bioinformatic investigations on *ppzV*. We carried out a protein-fold recognition search using the Phyre server [33]. Unexpectedly, this showed that PpzV possesses protein-fold homology to the TetR family of transcriptional regulators. Members of the TetR-family, e.g., PsrA [34], are involved in the regulation of phenazine biosynthesis in *Pseudomonas* and in the regulation of the biosynthesis of many secondary metabolites in *Streptomyces*. It therefore appears likely that PpzV is a new member of the TetR transcriptional regulators, despite its low sequence similarity to previously characterized members of this group. Based on the results of our inactivation experiments, it is tempting to speculate that PpzV is a positive regulator of the transcription of the prenyltransferase gene *ppzP* and/or of the mevalonate pathway genes.

## Conclusion

Our study showed that the endophenazine biosynthetic gene cluster comprises the 27 kb DNA region stretching from the phenazine prenyltransferase gene *ppzP* to the ABC transporters *ppzR1* and *ppzR2*. Inactivation experiments provided initial genetic insights into the regulation of phenazine biosynthesis in *Streptomyces*. While the complicated network responsible for the regulation of phenazine biosynthesis in *Pseudomonas* has been investigated in some detail [9,21,34–35], in *Streptomyces* none of the regulatory genes involved in the biosynthesis of phenazines or prenylated phenazines have been identified previously. Our mutational study now shows the involvement of several regulatory genes in this pathway. The gene *ppzV*, coding for a new type of TetR-related regulator, is likely to regulate the prenylation of the phenazine core. The gene *ppzY*, coding for a LysR-type regulator, appears to regulate the biosynthesis of the phenazine core, although further studies are required to confirm its precise role. In contrast, *orf3*, which codes for a protein similar to transcriptional modulators of the PemK-like family, is not required for endophenazine biosynthesis and is apparently situated outside of the endophenazine biosynthetic gene cluster. Our inactivation experiments identified *ppzM* as the first phenazine *N*-methyltransferase gene investigated in *Streptomyces*. We identified a new phenazine natural product, endophenazine E, and elucidated its structure. Endophenazine E represents a conjugate between the prenylated phenazine, endophenazine A, and L-glutamine.

## Experimental

### 

#### Bacterial strains, plasmids, and culture conditions

*S. anulatus* 9663 was isolated previously from the gut of a wood louse [22]. It was grown in liquid YMG medium [36] or on solid MS medium [36]. For the production of secondary metabolites, the medium described by Sedmera et al. [37] was used. *Escherichia coli* XL1 Blue MRF, *E. coli* SURE (Stratagene, Heidelberg, Germany), *E. coli* BW 25113, and *E. coli* ET 12567 (pUB307) were used for cloning and were grown in liquid or on solid (1.5% agar) Luria-Bertani or SOB medium at 37 °C. The REDIRECT technology kit for PCR targeting was obtained from Plant Bioscience Limited (Norwich, UK). For inactivation experiments, the *aac*(3)IV/*ori*T (apramycin resistance) cassette from pIJ773 [38] was used. Carbenicillin (50–100 μg·mL^−1^), apramycin (50 μg·mL^−1^), kanamycin (50 μg·mL^−1^), chloramphenicol (25 μg·mL^−1^), and nalidixic acid (20 μg·mL^−1^) were used for the selection of recombinant strains.

#### Chemicals

Kanamycin and carbenicillin were purchased from Genaxxon BioSciences GmbH (Biberach, Germany) and phenazine 1-carboxylic acid was from InFormatik. IPTG, Tris, NaCl, glycerol, dithiothreitol, MgCl_2_, formic acid, sodium dodecyl sulfate, polyacrylamide, and EDTA were from Carl Roth, Karlsruhe, Germany. Apramycin, nalidixic acid, methanol, Tween 20 and imidazole were from Sigma Aldrich, Steinheim, Germany. Merck supplied chloramphenicol, dipotassium hydrogen phosphate, potassium dihydrogen phosphate, sodium carbonate, sodium hydrogen carbonate and β-mercaptoethanol. Lysozyme was from Boehringer Ingelheim, Heidelberg, Germany.

#### Genetic procedures

Standard methods for DNA isolation and manipulation were performed as described by Kieser et al. [36] and Sambrook et al. [39]. DNA fragments were isolated from agarose gels by using a PCR purification kit (Amersham Biosciences). Genomic DNA was isolated by lysozyme treatment and phenol/chloroform extraction as described by Kieser et al. [36]. The construction, screening and heterologous expression of the phenazine biosynthetic gene cluster from *S. anulatus* was previously described [11].

#### Construction of cosmids ppzOS10, ppzOS11, ppzOS21-28

An apramycin resistance cassette (*aac(3)IV*) was amplified from plasmid pUG019 [40] using the corresponding primers mentioned in Table S2. The resulting 1077-bp PCR product was used to replace the desired genes on cosmid ppzOS04 by λ-RED-mediated recombination. Deletion of the *aac(3)IV* cassette from the resulting cosmids was carried out by using FLP-recombinase [41]. The resulting constructs were introduced into *S. coelicolor M512* by triparental mating [36].

#### Production and analysis of secondary metabolites

Culture methods and analysis techniques were adapted from Saleh et al. [11]. For the culture in Erlenmyer flasks, exconjugants of all mutants as well as wild type *S. anulatus* were precultured for 48 h in liquid YMG medium (50 mL). Then, 50 mL of production medium was inoculated with 2.5 mL of the precultures. The flasks were agitated on a rotary shaker at 30 °C and 200 rpm for 72–120 h.

For isolation of endophenazine A and endophenazine E, mycelia from 50 mL cultures were centrifuged at 3500 × *g* for 10 min. The cells were extracted with methanol (10 mL) by vortexing. The extract was mixed with sodium acetate buffer (10 mL; 1 M, pH 4.0) and extracted with dichloromethane (5 mL). After separation of the organic phase, the solvent was evaporated, and the residue was redissolved in methanol (0.5 mL). For the extraction of nonprenylated phenazines, the supernatant of the 50 mL cultures was adjusted to pH 4.0 by using 1 M HCl and extracted with 50 mL ethylacetate. After vortexing, the organic solvent was evaporated and the residue was dissolved in 500 µL methanol.

For the production in 24 square deep-well plates, homogenized and frozen inoculums, as described by Siebenberg et al. [14], were prepared from each mutant, inoculated into 3 mL of production media in each deep well and incubated for five days at 30 °C. For the analysis of the secondary metabolites, 1 mL was extracted as described above and analyzed by HPLC. For the analysis of the secondary metabolite profile over time, 100 µL from each deep well was collected after three, five and seven days and extracted as described above.

The extracts from supernatant and from mycelia were analyzed with HPLC (Agilent 1200 series; Waldbronn, Germany) by using an Eclipse XDB-C18 column (4.6 × 150 mm, 5 μm; Agilent) at a flow rate of 1 mL·min^−1^ with a linear gradient from 40 to 100% of solvent B in 20 min (solvent A: water/formic acid (999:1); solvent B, methanol) and detection at 252 and 365 nm. Additionally, a UV spectrum from 200 to 400 nm was logged by a photodiode array detector. The absorbance at 365 nm was used for quantitative analysis, employing an authentic reference sample of PCA as the external standard.

#### Analysis by LC–MS

The extracts were examined with LC–MS and LC–MS^2^ analysis by using a Nucleosil 100-C_18_ column (2 × 100 mm, 3 µm) coupled to an ESI mass spectrometer (LC/MSD Ultra Trap System XCT 6330; Agilent Technology). Analysis was carried out at a flow rate of 0.4 mL·min^−1^ with a linear gradient from 10 to 100% of solvent B in 15 min (solvent A: water/formic acid (999:1); solvent B: acetonitrile/formic acid (999.4:0.6)). Detection was carried out at 230, 260, 280, 360, and 435 nm. Electrospray ionization (positive and negative ionization) in Ultra Scan mode with capillary voltage of 3.5 kV and drying gas temperature of 350 °C was used for LC–MS analysis. For LC–MS^2^ and LC–MS^3^, the analysis was carried out in positive ionization mode with a capillary voltage of 3.5 kV at 350 °C.

#### Preparative isolation of endophenazine E

The strain *S. coelicolor*(ppzOS04) was precultured in 500 mL production medium for 48 h at 27 °C. This culture was inoculated into a 10 L fermenter containing the same production media and grown at 27 °C for 144 h. The cultures were then filtrated under vacuum by using 3% celite. The mycelia supernatant was discarded and the mycelia was extracted with methanol/acetone (1:1). The extract was concentrated in vacuo to an aqueous residue, adjusted to pH 4.0 by using HCl and extracted with ethyl acetate. The ethyl acetate extract was first treated with petrol ether. After evaporation, the extract residue was fractioned by using a liquid chromatography system with a Sephadex LH20 column (2.5 × 90 cm) and methanol as the mobile phase. The fractions containing endophenazine E as the main product were pooled and the solvents were evaporated. The residue was redissolved in methanol and applied to a preparative HPLC system with Reprosil Basic C18 column (250 × 20 mm). The separation was carried out with a linear gradient from 70 to 85% of solvent B in 15 min (solvent A: water/formic acid (999:1); solvent B: methanol) and the fractions containing pure endophenazine E were pooled and dried by lyophilisation, resulting in 50 mg of pure endophenazine E being extracted.

#### Identification of the stereochemical configuration of endophenazine E

The configuration of the amino acid glutamine in the structure of PCA-Gln was determined as described by Lämmerhofer and Lindner [16], by using an enantioselective HPLC system with two complementary chiral columns, which contained either quinine (QN) or quinidine (QD) derivatives as chiral selectors. To produce reference substances, 5 mg *N*,*N′*-dicyclohexylcarbodiimide (DCC) dissolved in 10 µL acetonitrile was added to 1 mg endophenazine A in 200 µL acetonitrile. The tube was heated to 60 °C for 1 h. Then, 2 mg *N*-hydroxysuccinimide, dissolved in 10 µL acetonitrile, was added to the reaction. The mixture was kept at 60 °C for 24 h. After being cooled to room temperature, acetonitrile was added to give a final volume of 300 µL. To a 100 µL aliquot of this solution, 1 mg D-glutamine in 100 µL carbonate buffer (0.1 M NaHCO_3_/0.1 M Na_2_CO_3_; 2:1 (v/v)) was added. To a further aliquot of 100 µL, 1 mg L-glutamine in 100 µL carbonate buffer was added. To a third aliquot of 50 µL, 50 µL carbonate buffer without glutamine was added. All three tubes were kept at 25 °C for three days and subsequently air dried at 25 °C. The residues were taken up in 100 µL MeOH and analysed by HPLC by using Chiralpak QN-AX and Chiralpak QD-AX columns (5 µm, 150 × 4 mm ID) (Chiral Technologies Europe, Illkirch, France). A mixture of methanol/acetic acid/ammonium acetate (99:1:0.25 (v/v/w)) was used as the mobile phase with an isocratic flow rate of 1 mL·min^−1^ and a column temperature of 25 °C. UV detection was carried out at 220, 250, 350 and 370 nm. On the QN-AX and QD-AX columns, the isolated compound PCA-Gln showed retention times of 9.3 min and 7.5 min, respectively, identical to the reference compound synthesized from L-Gln.

#### Overexpression and purification of PpzM Protein

Analogous to the method used for PpzP protein by Saleh et al. [11], the gene *ppzM* was amplified by using the primers *ppzM*_pHis_F (5′- CCG CCC ATG AGG AGA GGA TCC ATG AGT ACC GAC ATC GCA C-3′) and *ppzM*_pHis_R (5′-GTC GCC GGC CGT CGG CAC CTC GAG GTC AGC CGG CCG GGG TCA GG -3′). The underlined letters represent BamHI and XhoI restriction sites, respectively. The resulting PCR fragment was digested with BamHI and XhoI and ligated into plasmid pHis8 [42], digested with the same restriction enzymes. The resulting plasmid, pHis8-OS03, was verified by restriction mapping and sequencing. *E. coli* BL21(DE3)pLysS cells harbouring plasmid pHis8-OS03 were cultivated in 2 L of liquid TB medium containing 50 μg·mL^−1^ kanamycin and grown at 37 °C to an *A*_600_ of 0.6. The temperature was lowered to 20 °C, and isopropyl-1-thio-β-D-galactopyranoside was added to a final concentration of 0.5 mM. The cells were cultured for a further 10 h at 20 °C and harvested. Then, 30 mL of lysis buffer (50 mM Tris-HCl, pH 8.0, 1 M NaCl, 10% glycerol, 10 mM β-mercaptoethanol, 20 mM imidazole, 0.5 mg·mL^−1^ lysozyme, 0.5 mM phenylmethylsulfonyl fluoride) was added to the pellet (40 g). After being stirred at 4 °C for 30 min, cells were ruptured with a Branson sonifier at 4 °C. The lysate was centrifuged (55,000 × *g*, 45 min). Affinity chromatography with 4 mL of Ni^2+^-nitrilotriacetic acid-agarose resin (Qiagen, Hilden, Germany) was carried out according to the manufacturer's instructions, by using 2 × 2.5 mL of 250 mM imidazole (in 50 mM Tris-HCl, pH 8.0, 1 M NaCl, 10% glycerol, 10 mM β-mercaptoethanol) for elution. Subsequently, a buffer exchange was carried out by PD10 columns (Amersham Biosciences), which were eluted with 50 mM Tris-HCl, pH 8.0, 1 M NaCl, 10% glycerol, and 2 mM 1,4-dithiothreitol. Approximately 30 mg of purified PpzM was obtained from 2 L of cultures.

## Supporting Information

File 1 Analytical data, complete list of genes in cosmid ppzOS04, and PCR primers.

## References

[1] Laursen J B, Nielsen J (2004). Chem Rev.

[2] Mavrodi D V, Bonsall R F, Delaney S M, Soule M J, Phillips G, Thomashow L S (2001). J Bacteriol.

[3] Mavrodi D V, Blankenfeldt W, Thomashow L S (2006). Annu Rev Phytopathol.

[4] Ahuja E G, Janning P, Mentel M, Graebsch A, Breinbauer R, Hiller W, Costisella B, Thomashow L S, Mavrodi D V, Blankenfeldt W (2008). J Am Chem Soc.

[5] McDonald M, Mavrodi D V, Thomashow L S, Floss H G (2001). J Am Chem Soc.

[6] Mavrodi D V, Peever T L, Mavrodi O V, Parejko J A, Raaijmakers J M, Lemanceau P, Mazurier S, Heide L, Blankenfeldt W, Weller D M (2010). Appl Environ Microbiol.

[7] Pierson L S, Pierson E A (2010). Appl Microbiol Biotechnol.

[8] Girard G, van Rij E T, Lugtenberg B J J, Bloemberg G V (2006). Microbiology (Reading, U K).

[9] Girard G, Barends S, Rigali S, van Rij E T, Lugtenberg B J J, Bloemberg G V (2006). J Bacteriol.

[10] Ge Y, Yang S, Fang Y, Yang R, Mou D, Cui J, Wen L (2007). FEMS Microbiol Lett.

[11] Saleh O, Gust B, Boll B, Fiedler H P, Heide L (2009). J Biol Chem.

[12] Seeger K, Flinspach K, Haug-Schifferdecker E, Kulik A, Gust B, Fiedler H P, Heide L (2011). Microb Biotechnol.

[13] Haagen Y, Glück K, Fay K, Kammerer B, Gust B, Heide L (2006). ChemBioChem.

[14] Siebenberg S, Bapat P M, Lantz A E, Gust B, Heide L (2010). J Biosci Bioeng.

[15] Gomez-Escribano J P, Bibb M J (2011). Microb Biotechnol.

[16] Lämmerhofer M, Lindner W, Grinberg N, Grushka E (2008). Liquid Chromatographic Enantiomer Separation and Chiral Recognition by Cinchona Alkaloid-Derived Enantioselective Separation Materials. Advances in Chromatography.

[17] Gilpin M L, Fulston M, Payne D, Cramp R, Hood I (1995). J Antibiot.

[18] Schulz D, Nachtigall J, Riedlinger J, Schneider K, Poralla K, Imhoff J F, Beil W, Nicholson G, Fiedler H-P, Süssmuth R D (2009). J Antibiot.

[19] Masuda Y, Miyakawa K, Nishimura Y, Ohtsubo E (1993). J Bacteriol.

[20] Maddocks S E, Oyston P C F (2008). Microbiology (Reading, U K).

[21] Lu J, Huang X, Li K, Li S, Zhang M, Wang Y, Jiang H, Xu Y (2009). J Biotechnol.

[22] Gebhardt K, Schimana J, Krastel P, Dettner K, Rheinheimer J, Zeeck A, Fiedler H-P (2002). J Antibiot.

[23] Greenhagen B T, Shi K, Robinson H, Gamage S, Bera A K, Ladner J E, Parsons J F (2008). Biochemistry.

[24] Parsons J F, Greenhagen B T, Shi K, Calabrese K, Robinson H, Ladner J E (2007). Biochemistry.

[25] Okamura E, Tomita T, Sawa R, Nishiyama M, Kuzuyama T (2010). Proc Natl Acad Sci U S A.

[26] Kuzuyama T, Seto H (2003). Nat Prod Rep.

[27] Yang W, Ni L, Somerville R L (1993). Proc Natl Acad Sci U S A.

[28] Grandori R, Khalifah P, Boice J A, Fairman R, Giovanielli K, Carey J (1998). J Biol Chem.

[29] Lombó F, Braña A F, Salas J A, Méndez C (2004). ChemBioChem.

[30] Winter J M, Moffitt M C, Zazopoulos E, McAlpine J B, Dorrestein P C, Moore B S (2007). J Biol Chem.

[31] Kawasaki T, Hayashi Y, Kuzuyama T, Furihata K, Itoh N, Seto H, Dairi T (2006). J Bacteriol.

[32] Novakova R, Homerova D, Feckova L, Kormanec J (2005). Microbiology (Reading, U K).

[33] Kelley L A, Sternberg M J E (2009). Nat Protoc.

[34] Chin-A-Woeng T F C, van den Broek D, Lugtenberg B J J, Bloemberg G V (2005). Mol Plant-Microbe Interact.

[35] Heeb S, Haas D (2001). Mol Plant-Microbe Interact.

[36] Kieser T, Bibb M J, Buttner M J (2000). Practical Streptomyces genetics.

[37] Sedmera P, Pospíšil S, Novák J (1991). J Nat Prod.

[38] Gust B, Chandra G, Jakimowicz D, Yuqing T, Bruton C J, Chater K F (2004). Adv Appl Microbiol.

[39] Sambrook J, Russell D W (2001). Molecular Cloning. A Laboratory Manual.

[40] Eustáquio A S, Gust B, Galm U, Li S-M, Chater K F, Heide L (2005). Appl Environ Microbiol.

[41] Gust B, Challis G L, Fowler K, Kieser T, Chater K F (2003). Proc Natl Acad Sci U S A.

[42] Jez J M, Ferrer J-L, Bowman M E, Dixon R A, Noel J P (2000). Biochemistry.

