# Supplementary material for: Mutational analysis of a phenazine biosynthetic gene cluster in Streptomyces anulatus 9663
Source: Beilstein J Org Chem. 2012 Apr 4;8:501–13. doi: 10.3762/bjoc.8.57 (PMC3326630; doi:10.3762/bjoc.8.57)
Supplement: File 1 — Analytical data, complete list of genes in cosmid ppzOS04, and PCR primers. [file Beilstein_J_Org_Chem-08-501-s001.pdf]

Supporting Information

for

Mutational analysis of a phenazine biosynthetic gene cluster in *Streptomyces anulatus* 9663

Orwah Saleh[1], Katrin Flinspach[1], Lucia Westrich[1], Andreas Kulik[2], Bertolt Gust[1], Hans-Peter Fiedler[2] and Lutz Heide[*1]

Address: [1]Pharmaceutical Institute, University of Tübingen, Auf der Morgenstelle 8, 72076 Tübingen, Germany and [2]Institute of Microbiology and Infection Medicine, University of Tübingen, Auf der Morgenstelle 28, 72076 Tübingen, Germany

Email: Lutz Heide - heide@uni-tuebingen.de

[*]Corresponding author

[§]Fax: +497071295250

Analytical data, complete list of genes in cosmid ppzOS04, and PCR primers

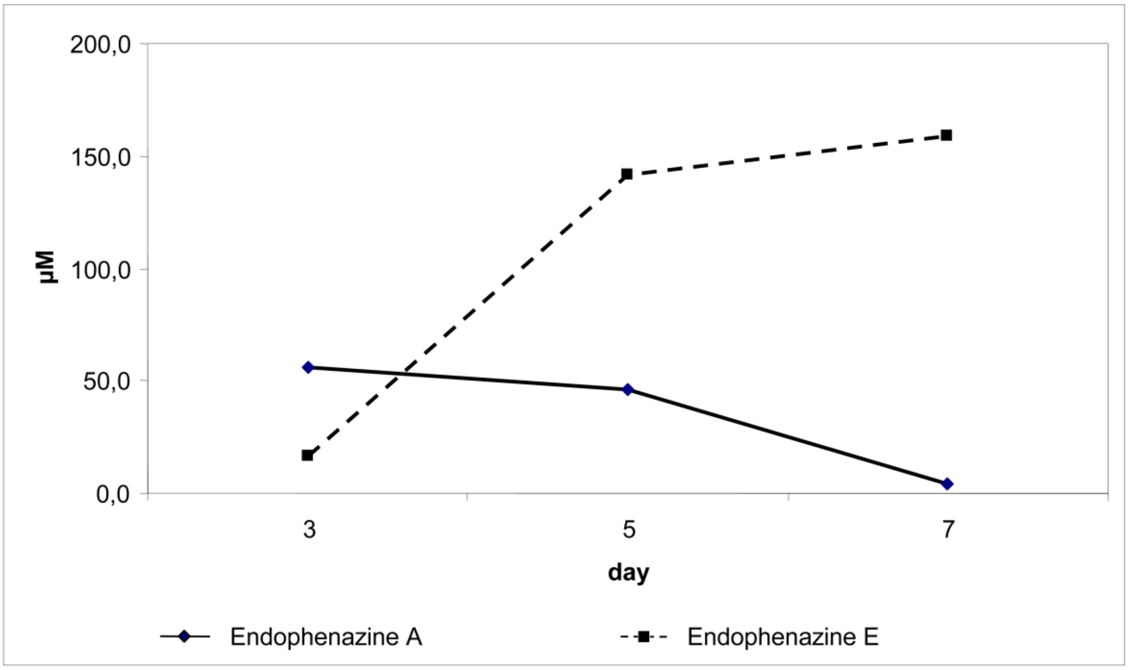

Figure S1: Production of endophenazine A and endophenazine E by *S. coelicolor* M512(ppzOS04) after cultivation for 3, 5 and 7 days in 24 deep-well plates.

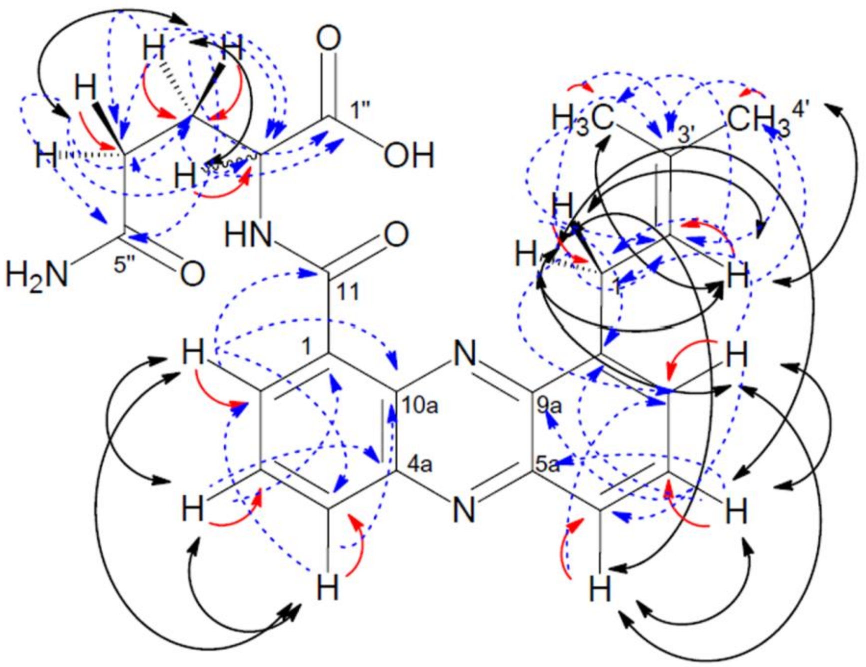

Figure S2: ^{1}H-^{1}H COSY (↔), HSQC (→) and HMBC (-----) correlations from 2D NMR experiments for endophenazine E.

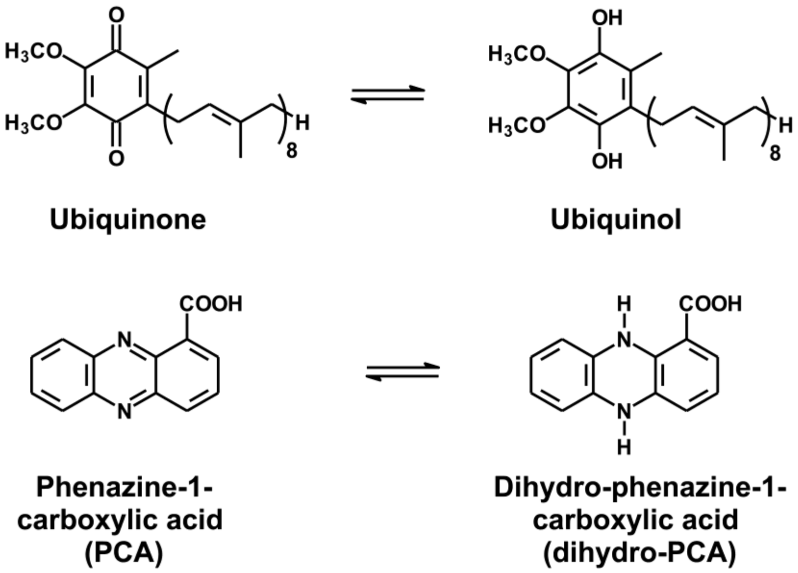

Figure S3: Reduction of ubiquinone to ubiquinol, catalyzed by cytochrome d ubiquinol oxidase, and reduction of phenazine-1-carboxylic acid to dihydro-phenazine-1-carboxylic acid, possibly catalyzed by PpzZ1/PpzZ2 of the endophenazine biosynthetic gene cluster of *Streptomyces anulatus* 9663.

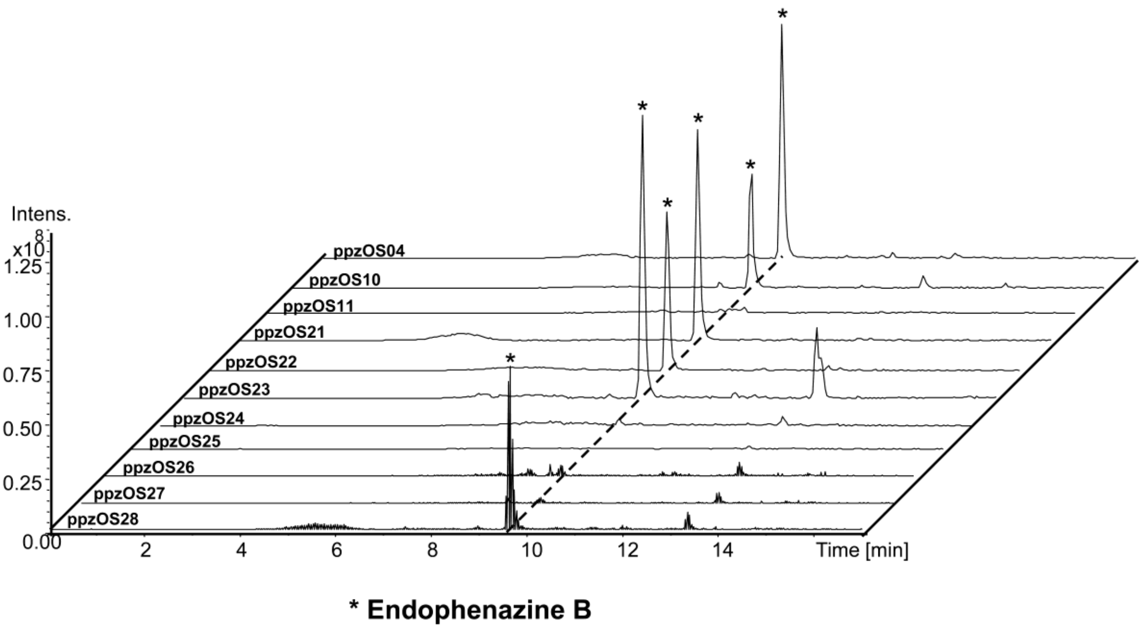

Figure S4: Extracted ion chromatograms for the mass of endophenazine B ($[M + H]^+ = 323$) for the heterologous host *S. coelicolor* M512 with the cosmid ppzOS04 carrying the intact phenazine biosynthetic gene cluster from *S. anulatus* and the heterologous host with the constructs ppzOS10-11 and ppzOS21-28.

Table S1: Genes identified on cosmid ppzOS04 from *S. anulatus* 9663.

Gene	aa	Proposed function	Orthologue identified by BLAST search	Identity/ Similarity %	Acc. Number
orf1	333	serine protease	putative serine protease, *Streptomyces roseosporus* NRRL 15998	93/98	ZP_04708306
orf2	342	aspartate-semialdehyde dehydrogenase	ASD2, *Streptomyces griseus* subsp. griseus NBRC 13350	97/99	YP_001826399
orf3	115	putative transcriptional modulator	*Streptomyces roseosporus* NRRL 11379	71/87	ZP_04708308
orf4	870	aminopeptidase N	*Streptomyces griseus* subsp. griseus NBRC 13350	98/99	YP_001826397
ppzP	299	prenyltransferase	EpzP, *Streptomyces cinnamonensis*	57/71	ADQ43372
mk	345	mevalonate kinase	MK, *Streptomyces cinnamonensis*	76/87	ADQ43373
mdpd	351	diphosphomevalonate decarboxylase	*Streptomyces* sp. CL190	77/84	BAB07791
pmk	371	phosphomevalonate kinase	*Streptomyces* sp. CL190	72/80	BAB07792
ippi	363	isopentenyl-diphosphate delta-isomerase	*Streptomyces* sp. CL190	79/88	Q9KWG2
hmgr	353	3-hydroxy-3-methylglutaryl coenzyme A reductase	HMGR, *Streptomyces cinnamonensis*	89/94	ADQ43377
hmgs	391	3-hydroxy-3-methylglutaryl CoA synthase	LPMA, *Streptomyces* sp. SN-1061M	85/92	ADC96649
ppzT	327	putative acetoacetyl-CoA synthase	*Streptomyces* sp. KO-3988	79/87	BAD86806
ppzU	221	flavodoxin	flavoprotein WrbA, *Streptomyces violaceusniger* Tü 4113	63/79	YP_004814680
ppzV	206	putative TetR-family regulator	EpzV, *Streptomyces cinnamonensis*	64/76	ADQ43382
ppzM	340	N-methyltransferase	EpzM, *Streptomyces cinnamonensis*	77/86	ADQ43384
ppzA	168	phenazine biosynthesis	EpzA2, *Streptomyces cinnamonensis*	83/88	ADQ43385
ppzG	233	FMN-dependent oxidase	EphzG, *Streptomyces cinnamonensis*	68/77	CAL34111
ppzF	279	*trans* 2,3-dihydro 3-hydroxyanthranilate isomerase	EpzF, *Streptomyces cinnamonensis*	87/94	ADQ43387
ppzE	646	2-amino-2-desoxy-isochorismate synthase	EphzE, *Streptomyces cinnamonensis*	82/87	CAL34110
ppzD	207	2,3-dihydro-3-hydroxy-anthranilate (DHHA) synthase	EphzD, *Streptomyces cinnamonensis*	85/92	CAL34109
ppzC	392	3-deoxy-D-arabino-heptulosonic acid 7-phosphate synthase	EphzC, *Streptomyces cinnamonensis*	82/89	CAL34108
ppzB	162	phenazine biosynthesis	EphzB, *Streptomyces cinnamonensis*	83/88	CAL34107
ppzK	419	FAD-dependent oxidoreductase	FAD-dependent pyridine nucleotide-disulfide oxidoreductase	55/66	YP_295688
ppzL	107	ferredoxin	ferredoxin, *Rhodopseudomonas palustris* HaA2	55/68	YP_487220
ppzY	290	transcriptional regulator	transcriptional regulator, *Streptomyces lividans* TK24	61/74	ZP_06530228

ppzZ1	430	cytochrome d ubiquinol oxidase, subunit I	cytochrome d ubiquinol oxidase, subunit I, *Stackebrandtia nassauensis* DSM 44728	54/66	YP_003509915
ppzZ2	344	cytochrome d ubiquinol oxidase, subunit II	cytochrome d ubiquinol oxidase, subunit II, *Stackebrandtia nassauensis* DSM 44728	48/60	YP_003509914
ppzR1	563	ABC transporter	cysteine ABC transporter permease, *Thermobispora bispora* DSM 43833	55/68	YP_003653341
ppzR2	585	ABC transporter	cysteine ABC transporter permease, *Streptosporangium roseum* DSM 43021	52/65	YP_003338959
orf12	384	allantoicase	putative allantoicase, *Streptomyces griseus* subsp. griseus NBRC 13350	98/99	YP_001826394

Table S2: Sequences of the primers used to construct the cosmids ppzOS10, ppzOS11 and ppzOS21-28.

Cosmid	Primer	Sequence
ppzOS10	18A9_L_for	5´-GCC AAG CAG GGC AAG CCC GCC CAC GAG TTC TCG GTG AAG TCT AGA ATT CCG GGG ATC CGT CGA CC-3´
	18A9_L_rev	5´-CTA CGC GGT GGC CGC CGC CGC GTC CGC CGC CCG GGC CTT ACT AGT TGT AGG CTG GAG CTG CTT C-3´
ppzOS11	18A9_R_for	5´-CGG AAT TCG TTA CTC CAG TCG CGT GCG TGT GTC GTC GGT TCT AGA ATT CCG GGG ATC CGT CGA CC-3´
	18A9_R_rev	5'-TTC GGC TGA GCT GAA AGA TAT CTG CGA AAA CTT TCG ATG ACT AGT TGT AGG CTG GAG CTG CTT C-3´
ppzOS21	18A9_fOrf2_for	5´-GCC AAG CAG GGC AAG CCC GCC CAC GAG TTC TCG GTG AAG TCT AGA ATT CCG GGG ATC CGT CGA CC-3´
	18A9_fOrf2_rev	5´-CCG GAG CCG GAC ACC GCC TGG TAG GAG GCG ACG GTC AGC ACT AGT TGT AGG CTG GAG CTG CTT C-3´
ppzOS22	18A9_fOrf4_for	5´-CGG AAT TCG TTA CTC CAG TCG CGT GCG TGT GTC GTC GGT TCT AGA ATT CCG GGG ATC CGT CGA CC-3´
	18A9_fOrf4_rev	5´-CGC CAT GAT CGG GTG CGT GGA GGG CAG CTG GTC CTG CCG ACT AGT TGT AGG CTG GAG CTG CTT C-3´
ppzOS23	Knock_ppz2_F	5´-CCC CGT TTT CCC GTT CAC TTC TTC CCA GAG GGG GGC ATG TCT AGA ATT CCG GGG ATC CGT CGA CC-3´
	Knock_ppz2_R	5´-GCG CCA CAG GCG CCG GCC CCG GTG TCG TCT TGA CGG TCT ACT AGT TGT AGG CTG GAG CTG CTT C-3´
ppzOS24	Knock_ppz3_F	5´-GGC CGG CGC CTG TGG CGC CGG CCC CGG CCG AGA CCG ATG TCT AGA ATT CCG GGG ATC CGT CGA CC-3´
	Knock_ppz3_R	5´-ACG GCA CTC CGG GCC GCT CGG AGT TCG ACA TAG AGG AAC ACT AGT TGT AGG CTG GAG CTG CTT C-3´
ppzOS25	Knock_ppz4_F	5´-CAT CAC TGG AAC AGG TCA TCT CAT GAA GGA GAC TCC ATG TCT AGA ATT CCG GGG ATC CGT CGA CC-3´
	Knock_ppz4_R	5´-CCA CGT TCG TCG GCA CGA GTC TGT GGC GGG GCA GCC TGG ACT AGT TGT AGG CTG GAG CTG CTT C-3´
ppzOS26	Knock_ppzM_F	5´-CCC GCC CGC CCG CAC CCG CCC ATG AGG AGA CAG GCC ATG TCT AGA ATT CCG GGG ATC CGT CGA CC-3´
	Knock_ppzM_R	5´-GGC GGG AGG ATC CGG TCG CCG GCC GTC GGC ACG TCA TGG ACT AGT TGT AGG CTG GAG CTG CTT C-3´
ppzOS27	18A9_fppz8_F	5´-CCT CAC CGA CTT CCA AGC GCT GCT CAC CAA CTC CAG CGC TCT AGA ATT CCG GGG ATC CGT CGA CC-3´
	18A9_fppz8_R	5´-TTC GGC TGA GCT GAA AGA TAT CTG CGA AAA CTT TCG ATG ACT AGT TGT AGG CTG GAG CTG CTT C-3´
ppzOS28	18A9_fOrf5_F	5´-GCA TCA GGC CCG GGT CGT GGG CGA GAT GGC GGA CGC CCA TCT AGA ATT CCG GGG ATC CGT CGA CC-3´
	18A9_fOrf5_R	5´-TTC GGC TGA GCT GAA AGA TAT CTG CGA AAA CTT TCG ATG ACT AGT TGT AGG CTG GAG CTG CTT C-3´
